# Skin oncoplasties: O-to-Z technique a technique of choice in situation of limited resources? Case of Burkina Faso

**DOI:** 10.1186/s12957-022-02580-8

**Published:** 2022-04-07

**Authors:** Nayi Zongo, N. L. Marie Ouedraogo, Mamadou Windsouri, Laure S. C. Yameogo, Thierry R. Kouchika Chabi, Pascal Niamba, Adama Traore

**Affiliations:** 1 General and Oncology Surgery, Yalgado Ouedraogo Teaching Hospital, Ouagadougou, Burkina Faso; 2 Surgical Oncology, Joseph Ki-Zerbo University, Ouagadougou, Burkina Faso; 3 Saint Camille Hospital in Ouagadougou, Surgical Pole, Ouagadougou, Burkina Faso; 4 General Surgery, Tengandogo Teaching Hospital, Ouagadougou, Burkina Faso; 5 Dermatology, Yalgado Ouedraogo Teaching Hospital, Ouagadougou, Burkina Faso

**Keywords:** Cancer-skin, O-to-Z technique, Healing

## Abstract

**Background:**

In developing countries, the long delays in consultation lead to a delay in diagnosis and management of the skin tumors. The lesions are often large and bring the problem of skin coverage after their resections. Several reconstruction techniques allow skin coverage. The objective of this study is to describe the place of O-to-Z technique in the surgical treatment of skin cancers in Ouagadougou. We hypothesized that O-to-Z technique reduces healing times and the number of dressings compared with directed wound healing.

**Patients and methods:**

It was a two-center, retrospective, descriptive study on O-to-Z technique in skin cancers. It included patients who underwent surgery between January 1st, 2013 and March 30th, 2021 in Ouagadougou. Scar quality and healing time in Z-plasty were compared with those of secondary healing. We used the Student’s *t* test.

**Results:**

In 8 years and 3 months, 171 skin cancers were identified. The mean time to consultation was 13.6 months. The average size of the tumors was 9 cm. An O-to-Z technique was performed in 42 cases, being 58.3% of the patients operated on. The average healing time was 15 days. It was four and a half times shorter in O-to-Z technique than in secondary healing. Ischemic necrosis of the Z-corner was noted in 7 cases. The recurrence rate in O-to-Z technique and secondary healing was 7.1% and 9.1% respectively. Hypertrophic or keloidal scars were noticed in 7 cases and hypochromia in 2 cases.

**Conclusion:**

O-to-Z technique is a technique of choice for skin coverage after large resections in surgical oncology. It reduces the healing time and the cost of postoperative care without increasing the risk of tumor recurrence.

**Supplementary Information:**

The online version contains supplementary material available at 10.1186/s12957-022-02580-8.

## Introduction

Skin cancers are a public health problem. Their incidence has risen sharply in recent years worldwide, making them the third most common type of cancer [1]. One in three new cancers in Switzerland is a skin cancer [2]. They include basal cell carcinomas, squamous cell carcinomas, melanomas, and cutaneous sarcomas [3–5]. Surgery plays an important role in the management of skin cancers [3, 6, 7]. The modalities of surgical treatment depend on the location, histological type, size of the lesion, and the patient’s condition [6, 7]. In the West, the diagnosis of skin tumors is made at an early stage, with small lesions allowing resection and suturing in majority of cases [8–10]. The situation is quite different in developing countries, particularly in Burkina Faso [3, 7]. Indeed, the long delays in consultation lead to a delay in diagnosis and management [7]. The lesions are often large and bring the problem of skin coverage after their resections. Several reconstruction techniques allow skin coverage [10, 11]. Skin grafts require a good basement and good hygiene conditions. Secondary healing involves numerous wound dressings over several months. The mobilization of fascia-muscle flaps requires technical skills and a trained team [7, 10–13]. O-to-Z technique, a technique which has being used for long in plastic surgery, is less complex to perform and is therefore an alternative for skin coverage after large tumor resections [11, 13, 14]. In Burkina Faso, O-to-Z technique has been used for several years now, following skin cancers large resections [6]. The objective of this study is to describe its indications, technique and results in Ouagadougou.

## Patients and methods

This is a two-center, retrospective, descriptive study on O-to-Z technique in skin cancer, carried out between January 1st, 2013 and March 30th, 2021 in Ouagadougou. The surgical departments of the Yalgado Ouédraogo Teaching Hospital and the Schiphra Protestant Hospital were used as study sites. The study population was represented by all patients with a skin tumor who had undergone excision. Patients who had an O-to-Z technique after skin cancer surgery and were followed until healing were included in this study. Our data sources were the referral forms, the operating theatre register, and the medical records of the surgical department of the Yalgado Ouédraogo Teaching Hospital and the Schiphra Protestant Hospital. The data was collected using a data collection form. We took into account variables such as age, sex of patients, topography and size of tumors, histological type, closure technique after tumor resection, and complications. Data analysis was done by EPI info software. The patients were seen twice a week during the dressings and every 3 months for 2 years after the healing. Then, we see them every 6 months. After the surgery, during the controls, we looked for flap necrosis, tumor recurrence, which we always confirm by histological examination of recurrence biopsy specimens. The time of recurrence and their treatment were also noted. We also assessed the quality of the scar.

The average healing time in the group of patients who had an O-to-Z technique was compared to the average healing time in the group of patients who had a secondary wound healing. This comparison was made possible by using the Student’s *t* test. Our hypotheses were H0 = healing times are the same in both groups (secondary wound healing and O-to-Z technique) and H1: the delays are different. Data collection was authorized by the hospital directors and the heads of surgery and dermatology at these hospitals. Data collection was anonymous.

## Results

**Fig. 1:**
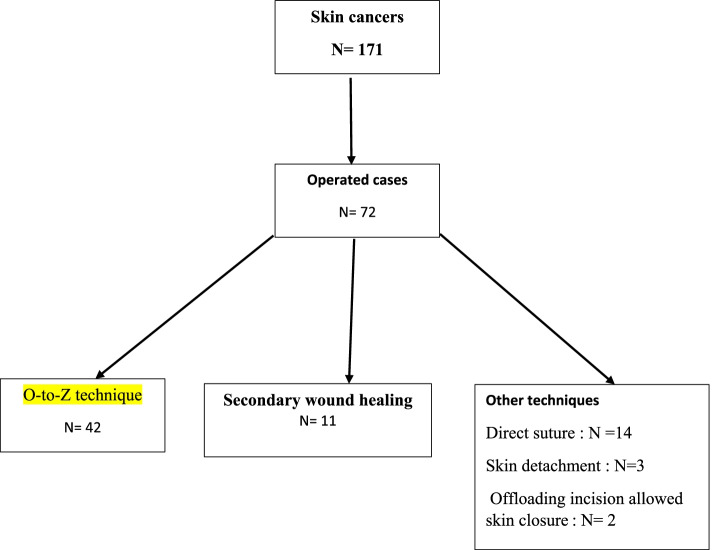
Flow chart

**Fig. 2:**
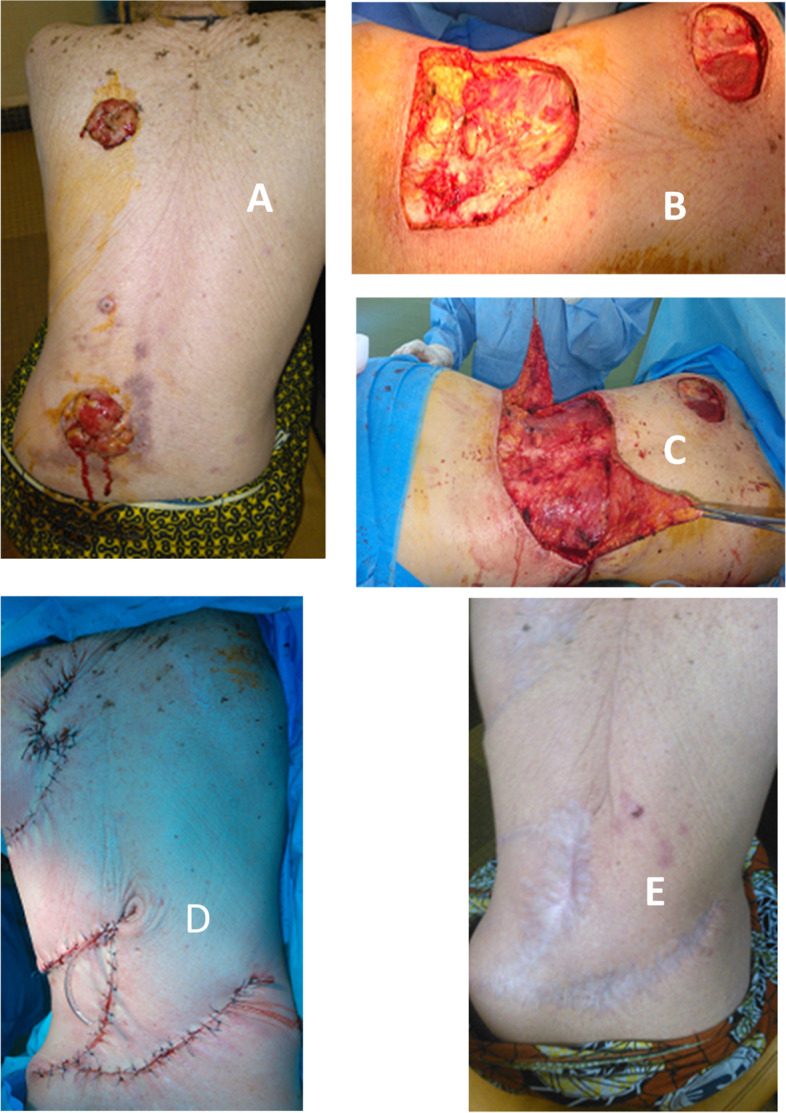
O-to-Z technique and multifocal carcinoma of the back in an albino. **A** Bifocal squamous cell carcinoma of the back. **B** Surgical wounds after tumor resection. **C** Mobilization of skin flaps. **D** Appearance after O-to-Z technique. **E** Scar appearance 1 year after O-to-Z technique surgery

**Fig. 3:**
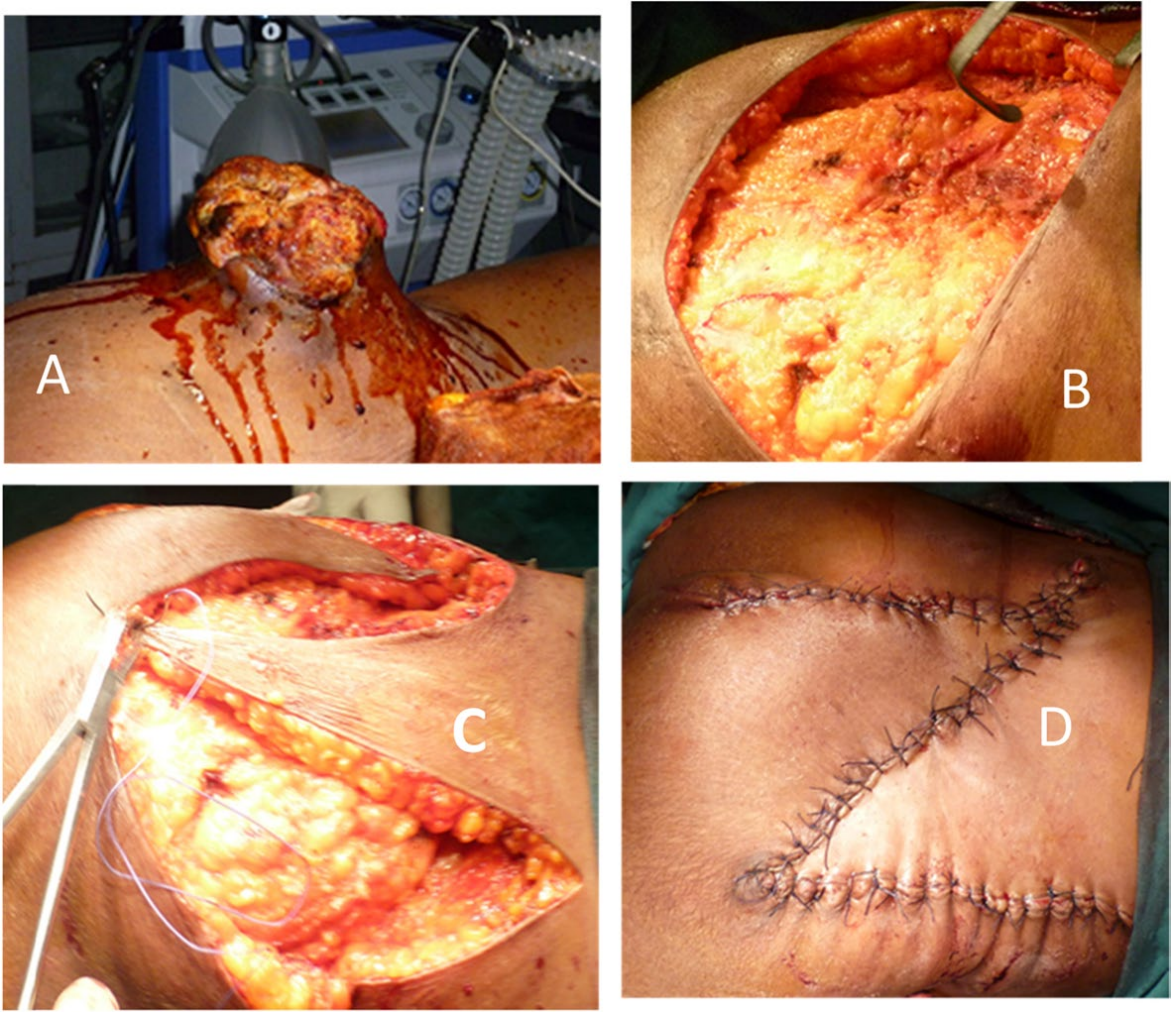
O-to-Z technique and skin sarcoma of the buttock. **A** Ulcerative burgeoning skin tumor of the right buttock. **B** Operative wound after tumor resection. **C** Mobilization of skin flaps. **D** Appearance after O-to-Z technique allowing skin coverage of the wound

**Fig. 4:**
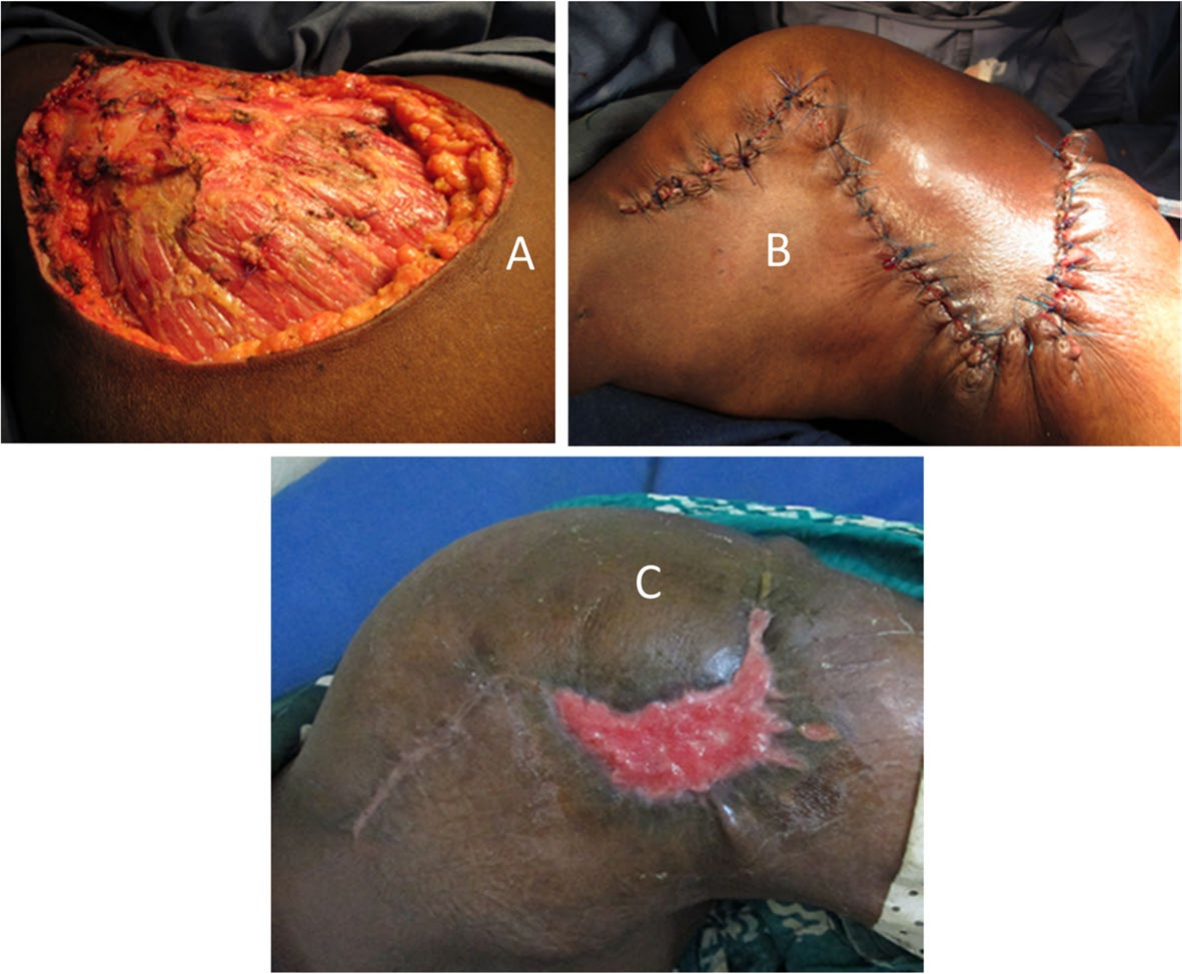
Corner necrosis after O-to-Z technique treated with rapture and directed healing. **A** Buttock wound after lumpectomy. **B** O-to-Z technique. **C** Necrotic Z-angle appearance after necrosectomy

In 8 years and 3 months, 171 skin cancers were identified. The average age of the patients was 48.5 years with extremes of 15 and 75 years (Table 1). Sixty percent of the patients were men. The average time to consultation was 13.6 months. The cancers were located on the thorax in 24% of cases (Table 1). The average size of the tumors was 9 cm, with a size greater than 5 cm in 63.3% of cases (Table 1). Cutaneous carcinomas accounted for 60%, melanomas for 25% and sarcomas for 15% of cases (Table 1). Surgery was performed on 72 patients (42%). After lumpectomy or compartmental surgery, direct suture, skin detachment, and offloading incision allowed skin closure in 14 cases (19.4%), 3 cases (4.2%), and 2 cases (2.8%) respectively. Secondary wound healing was the option in 11 cases (15.3%) with an average healing time of 67 ± 31 days with extremes of 52 and 126 days. On average, two dressings were made per week. Reconstructive surgery was performed for carcinomas and sarcomas. An O-to-Z technique was performed in 42 cases, being 58.3% of the patients (Figs. 1, 2, 3, and 4). The indication was a wide resection with an impossibility of direct suture. We only performed this plasty when the resection was at least macroscopically complete during surgery. The lateral margins of resection varied between 1.5 and 3 cm with an average of 2.4 cm in carcinomas (34 cases), 3 to 5 cm in sarcomas (7 cases). The length of the mobilized skin flaps was less than twice their width in all cases (Figs. 1 and 2). The average healing time was 15 ± 4.1 days. The fastest healing was noted after 12 days, and the latest healing was noted after 33 days. Dressings were made twice a week until healing was achieved. Partial flap necrosis was noticed in 2 cases. Ischemic necrosis of the “Z” angles was noticed in 5 cases (Fig. 3). Healing was achieved by excision of the necrotic tissue and further wound dressing. Histology of the surgical specimen referred to pathology noted an R0 resection in 40/42 cases. Incomplete resections were reoperated followed by directed healing.

**Table 1:** Clinico-pathological characteristics of patients who had a O-to-Z technique *n*=42

	Number	Percentage %
**Age of patients (years)**		
[15–25]	9	21
[25–50]	23	56
[50–75]	10	23
Total	42	100
**Topography of cancers**		
Thorax	10	24
Abdomen	8	19
Buttock	9	1
Thigh	7	17
Leg	6	14
Arm	2	5
Total	42	100
**Tumour size (cm)**		
[0–5]	4	10
[5–10]	17	40
[10–15]	11	25
[15–20]	6	15
More than 20	4	10
**Histological type**		
Darier and Ferrand Dermato-fibrosarcoma	2	4
Fibrosarcoma	4	10
Squamous cell carcinoma	29	72
Basal cell carcinoma	5	12
Undifferentiated sarcoma	2	4

**Fig. 5:**
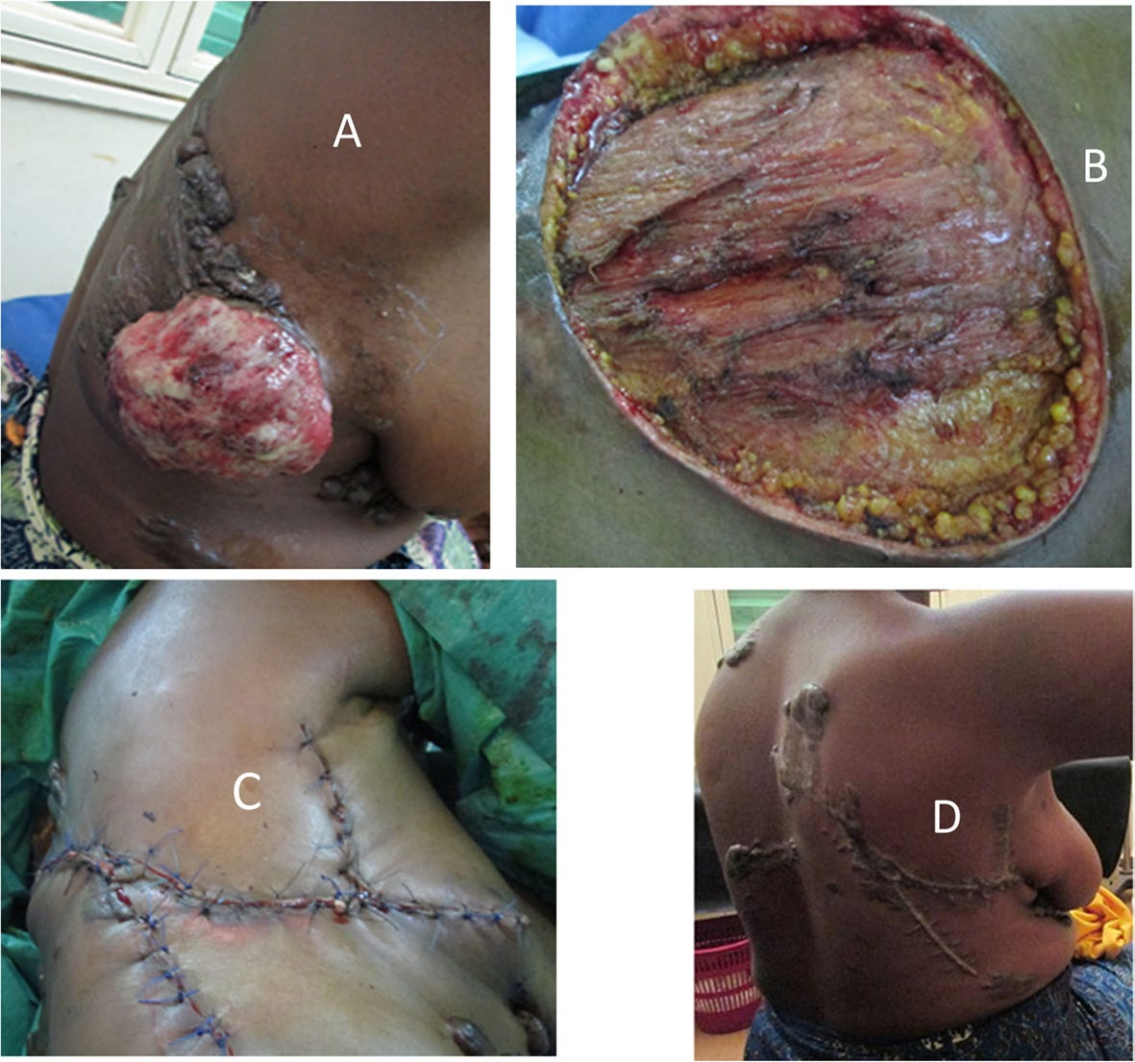
O-to-Z technique complicated by keloids. **A** Cutaneous sarcoma. **B** Wound after resection. **C** O-to-Z technique. **D** Keloid healing

Recurrence was observed in 3 cases. There were two patients operated on for a sarcoma (dermatofobrosarcoma of Darrier and Ferrand) and another operated on for a squamous cell carcinoma. The recurrences were all observed within 6 months after surgery. A wide resection was performed in these three cases of recurrence. The time required for secondary healing compared to Z-plasty was 4.5 times longer (Student’s *t* test, *t* = 14.21, *t* > − 2,101, *t* > 2, 101). The recurrence rate in O-to-Z technique and secondary healing was 7.1% and 9.1% respectively. Hypertrophic or keloidal scars were noticed in 7 cases (Figs. 4 and 5) and hypochromia in 2 cases (Fig. 1).

## Discussion

Skin cancers are increasing in incidence and are ranked third among all cancers [1, 15]. They are among the most common in the West [1, 15, 16]. In Australia, skin cancers have the highest incidence in the world with 33.6 cases/100,000 population [16]. This high frequency in the West contrasts with their relative rarity in Africa. Indeed, skin cancers represent 7.5% to 11.8% of all cancers in Africa [4, 17]. Skin cancers, although regularly diagnosed in Burkina Faso, remain relatively rare compared to the European and American literature [7]. Despite their low frequency, these cancers present difficulties in their management. Indeed, their diagnosis is late due to long delays in consultation (13.6 months) and consequently tumor sizes are large with an average of 9 cm. This contrasts with the small tumor sizes noted by some authors in developed countries, which range from 0.4 cm to 2 cm [8, 10]. When the tumor is small, direct excision-suture is possible [18]. However, when the tumor is large, the resection leaves large defects that can be filled by several procedures [16, 19]. There are many indications for skin coverage, including post-traumatic skin defects, surgical excision for benign or malignant tumors, burns, and deformities [18]. Skin coverage after large tumor resections remain a real challenge for healing. Direct suturing helped by the intrinsic elasticity and plasticity of the skin is no more possible [18, 19]. The prerequisite for direct suturing is an early diagnosis with small resections. This is far from being the case in our series where the average size of the tumors was 9 cm. Several methods of skin coverage must therefore be used [18, 19]. Pedicle flaps are used to fill in surgical defects [19]. Local skin or musculocutaneous T- and H-shaped flaps are used to treat skin defects in the forehead [20]. Rhombic flaps, which are local transposition flaps, are used to fill defects after skin cancer surgery in the head and neck region [19]. O-to-Z technique is the most commonly used technique in precarious situations and has solved 58.3% of the skin coverage problems of the trunk and limbs in our series. In addition to the size of the tumor, the indications for O-to-Z technique in our series were the absence of superinfection of the tumor, the absence of bony relief making it difficult to mobilize the flaps, and the localization of the tumor in an area where flaps can be mobilized. Given the small sample size and the limited number of surgeons who perform O-to-Z technique, it is difficult to draw conclusions, but clear trends emerged. Indeed, only 42 patients underwent O-to-Z technique. Although all cases were performed in the main city of the country, the application of this technique to the rest of the country should be possible with the same results because the practice conditions are similar. The size of the mobilized flaps remains function of the width of the surgical wound [18, 20]. However, for vitality of the flaps in the O-to-Z technique that are free, non-pediculized flaps, we followed the 2:1 rule, meaning the length should not be more than twice the width.

In a situation of limited resources, diagnostic delays, poor results and inaccessibility of chemotherapy, and the absence of radiotherapy give surgery a central place in the management of skin cancers. In case of large tumor sizes, the surgeon has the choice between directed healing and mobilization of skin flaps or skin grafts [19]. O-to-Z technique was performed in 58.3% of our patients with tumors size between 5 and 20 cm. O-to-Z technique allowed skin closure after large skin resections. Unlike vascularized flaps, it does not require a great technical skill, is fast to perform and accessible to most surgeons. The average healing time after O-to-Z technique was 15 days. Min and col. in their series found 29 days [13]. In our series, this healing time is 4 times longer in secondary healing. In addition, with an average of two wound dressings per week, it makes the total number of wound dressings to be 5 times higher in secondary wound healing than in O-to-Z technique.

The flap does not increase the recurrence rate, nor does it interfere with other adjuvant oncological treatments [19, 20]. The advantages of O-to-Z technique over secondary wound healing and skin grafting are short healing times and low postoperative care costs. O-to-Z technique thus seems to us to be a technique of choice in precarious situations for low-income countries such as Burkina Faso. In our series, the O-to-Z technique proved to be practicable, simple to perform and with very few complications. The healing time was short compared to secondary healing. It therefore reduces the number of dressings, trips to health centers, and the cost of care. These skin oncoplasties also reduce the rate of recurrence because of the large resections they allow the surgeon to perform without having to worry about compromising skin closure.

## Conclusion

Cutaneous oncoplastic surgery is in its onset in Burkina Faso. O-to-Z technique allows skin coverage while optimizing healing. It reduces the healing time compared to secondary healing and consequently the number of wound dressings and trips to health centers, in short the cost of care. It also reduces recurrence rates because of the large resections it allows without the surgeon having to worry about compromising skin closure. In addition to sarcomas and cutaneous carcinomas, it should also be used for rare skin cancers such as melanomas. The promotion of oncoplasty, a larger cohort and sufficient hindsight would allow a better appreciation of its advantages in the precarious situation of Burkina Faso.

## Supplementary Information

**Additional file 1.**

## Data Availability

The datasets used and/or analyzed during the current study are available from the corresponding author on reasonable request.
