# Supplementary material for: Skin oncoplasties: O-to-Z technique a technique of choice in situation of limited resources? Case of Burkina Faso
Source: World J Surg Oncol. 2022 Apr 7;20:113. doi: 10.1186/s12957-022-02580-8 (PMC8988396; doi:10.1186/s12957-022-02580-8)
Supplement: Supplementary file 1 — Additional file 1. [file 12957_2022_2580_MOESM1_ESM.docx]

**Data collection**

Name ..........................surname ...........................Contacts …… Phone number ..................

Sex: M F Age....................................Origin: urban rural

Occupation: civil servant housewife farmer trader student military student

Other: specify job.............................................................

Weight .................... Size .........................IMC.........................

Marital status ................................................................

**Reason for consultation:**

Emergency Symptoms Diagnosis already posed

Describe the picture …………………………………………………………………………

Date of consultation :.............................

Time of consultation..........................................................................................................................

Start of care ......................................................................................................................................

**History**

Medical..........................Surgical...........................................Familial........................................................

-Eating and lifesty

**Clinic**

-WHO score ...............................Clinical anaemia: yes no

-Tumour description (size and appearance) ...........................................................................................

-Superficial adenopathy: yes no

Topography..............................................................................

**Para clinical**

-Radiological profile and CT scan ................................. ......................................................

-Anatomopathology .................................................................. ...........................................

Preoperative check-up

Blood crase disorders Malnutrition Hypertension rhythm disorders

Anemia

**Techniques**

Z-plasty Direct suture Simple skin detachment incision Directed healing

Directed healing Other …………………………………………………………………………………

**Complications**

Suppuration loosening of the sutures Unsightly scarring Bleeding
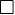
 Necrosis

**Chemotherapy**

Specify chemotherapy protocol .....................Duration of treatment ............................

Number of courses ...............Side effects ...........................................................................

Management of side effects .................................................................................................

**Radiotherapy**

Specify chemotherapy protocol ..............Duration of treatment .....................................................

Number of courses ..............................Side effects ..................................

Management of side effects ...................................................................................................

**Progression under treatment**

Death Continuation of cancer

Remission Recurrence
